# The Prognostic and Clinicopathological Significance of Systemic Immune-Inflammation Index in Bladder Cancer

**DOI:** 10.3389/fimmu.2022.865643

**Published:** 2022-04-28

**Authors:** Jinze Li, Dehong Cao, Yin Huang, Qiao Xiong, Daqing Tan, Liangren Liu, Tianhai Lin, Qiang Wei

**Affiliations:** ^1^ Department of Urology, Institute of Urology, West China Hospital, Sichuan University, Chengdu, China; ^2^ West China School of Clinical Medicine, Sichuan University, Chengdu, China

**Keywords:** bladder cancer, systemic immune-inflammation index, survival, prognosis, meta-analysis

## Abstract

**Background:**

Systemic immune-inflammation index (SII) has recently emerged as a biomarker for the prognosis of a variety of malignant tumors. However, the role of SII in bladder cancer (BC) remains unclear. To this end, we performed a pooled analysis to investigate the prognostic value of preoperative SII in patients with BC.

**Methods:**

A comprehensive search of electronic databases (PubMed/Medline, Web of Science, Scopus, and Cochrane Central Register of Controlled Trials) was conducted to determine the eligible studies that were published until January 2022. Pooled hazard ratios (HRs) and odds ratios (ORs) with 95% confidence intervals (CIs) were calculated to evaluate the association between preoperative SII and the prognosis and clinicopathological characteristics of BC.

**Results:**

Ten studies with 7,087 patients were included in this analysis. SII was observed to be correlated with inferior overall survival (HR = 1.22, 95% CI 1.04–1.44, p = 0.013), cancer-specific survival (HR = 1.68, 95% CI 1.14–2.47, *p* = 0.009), and recurrence-free survival (HR = 1.29, 95% CI 1.03–1.61, *p* = 0.027). An increased preoperative SII was also associated with poor tumor differentiation, higher tumor stage, presence of lymph node involvement, and tumor size ≥3 cm (all *p* < 0.05).

**Conclusions:**

An elevated preoperative SII is significantly associated with worse survival outcomes and adverse pathological features in patients with BC. Hence, SII may serve as a strong independent prognostic predictor for patients with BC after surgery.

## Introduction

Bladder cancer (BC) is one of the common urological malignancies, with an estimated 573,000 new BC cases and 213,000 deaths worldwide in 2020 (1). The disease could be divided into two subtypes, namely, non-muscle-invasive bladder cancer (NMIBC) and muscle-invasive bladder cancer (MIBC), according to different clinical progressions and prognoses. Approximately 75% of patients initially present with NMIBC, as NMIBC is associated with a lower risk of death and intravesical recurrence, the 5-year risk of recurrence ranges from 30% to 80%, even after transurethral resection of bladder tumor (TURBT) treatment (2, 3). In addition, despite the administration of postoperative adjuvant drug chemotherapy or immunotherapy, the 5-year overall survival (OS) rate for MIBC is approximately only 50%, and the risk of recurrence is high (4). Therefore, useful biomarkers should be sought in clinical practice to help diagnose, evaluate, and determine the prognosis of BC.

Accumulating evidence suggests that inflammatory responses in the tumor microenvironment (TME) might play a critical role in bladder tumorigenesis, proliferation, progression, and metastasis (5, 6). Inflammation can lead to leukocytosis, neutropenia, and thrombocytopenia. Therefore, various blood-based markers of inflammation, including neutrophil–lymphocyte ratio (NLR), platelet–lymphocyte ratio (PLR), and lymphocyte–monocyte ratio (LMR) have been explored to improve prognostic tools for BC risk stratification and outcomes (7). However, these markers integrate only two types of immune cells, and their predictive and prognostic capabilities are inadequate. The systemic immune-inflammation index (SII) is a novel index based on the count of neutrophils, lymphocytes, and platelets, which is reportedly correlated with survival outcomes for a variety of cancers (8).

Although previous studies have explored the prognostic value of SII in BC patients, the results were not consistent. SII was considered an important prognostic factor in some studies (9–11), whereas other studies have found no significant association between SII and survival outcomes of BC (12, 13). Therefore, this study aimed to use the available data to evaluate the prognostic role of preoperative SII in patients with BC and the association between SII and clinicopathological features of BC.

## Materials and Methods

### Protocol

This study was conducted following the 2020 Preferred Reporting Items for Systematic Reviews and Meta-Analyses (PRISMA) guideline (14) and was registered in PROSPERO (CRD42021274320, website link: https://www.crd.york.ac.uk/PROSPERO/display_record.php?RecordID=274320).

### Literature Search

We searched electronic databases including PubMed/Medline, Web of Science, Scopus, and Cochrane Central Register of Controlled Trials to determine the studies published from inception to January 2022 in the English language. A literature search was performed using the following search terms: (bladder cancer OR bladder neoplasms OR bladder tumor OR bladder carcinoma OR BC) AND (systemic immune-inflammation index OR SII). To supplement the identified citations, we also searched the list of references for relevant reviews and meta-analyses. Discrepancies were addressed through discussion or ultimately resolved by a third-party adjudication.

### Selection Criteria

Studies were included based on the following criteria: (i) patients had been histopathologically diagnosed with MIBC or NMIBC; (ii) hazard ratios (HRs) and corresponding 95% confidence intervals (CIs) for preoperative SII and survival outcomes, including OS, cancer-specific survival (CSS), and/or recurrence-free survival (RFS) had been reported or the relationship between SII and clinicopathological characteristics of BC had been obtained; and (iii) a cutoff value for preoperative SII had been stated. The categories of studies excluded were as follows: (i) basic research or animal studies; (ii) reviews, meta-analyses, comments, conference abstracts, case reports, and unpublished research; (iii) studies in which data were unavailable or insufficient; and (iv) duplicate publications.

### Data Extraction and Quality Assessment

The data from qualified publications were extracted by two evaluators separately, and discrepancies were discussed through negotiation or ultimately resolved by third-party adjudication. The data extracted constituted of the following: name of first author, year of publication, study area, study design, type of tumor, treatment, sample size, age of enrolled patients, cutoff value for SII, method of analysis, survival outcomes, and follow-up period. In addition, all of the survival outcomes were directly presented as HRs and corresponding 95% CIs. The primary outcome of this meta-analysis was the OS, and the secondary outcomes were CSS and RFS. Data from multivariate analysis were used when the data in a study had been analyzed in two ways simultaneously. The methodological quality of each included study was assessed using the Newcastle–Ottawa Scale (9 points highest score) (15). In this meta-analysis, we considered studies with a score from 7 to 9 to be of high quality.

### Statistical Analyses

Pooled HRs with 95% CIs were used to assess the association between preoperative SII and survival outcomes. Cochran’s Q and Higgin’s I^2^ tests were used to estimate heterogeneity among studies. I^2^ > 50% or *p* < 0.10 indicated significant heterogeneity. This meta-analysis used a random-effects model for pooled analysis. Moreover, subgroup analysis was conducted to explore potential sources of heterogeneity. Sensitivity analyses were also conducted to assess the effect of individual study data on survival outcomes. Pooled odds ratios (ORs) were used to identify the relationship between SII and clinicopathological factors, with 95% CIs calculated for both. Begg’s test was used for any potential publication biases. All statistical analyses were performed using Stata v. 15. *P*-value <0.05 was considered statistically significant.

## Results

### Study Characteristics

**Figure 1:**
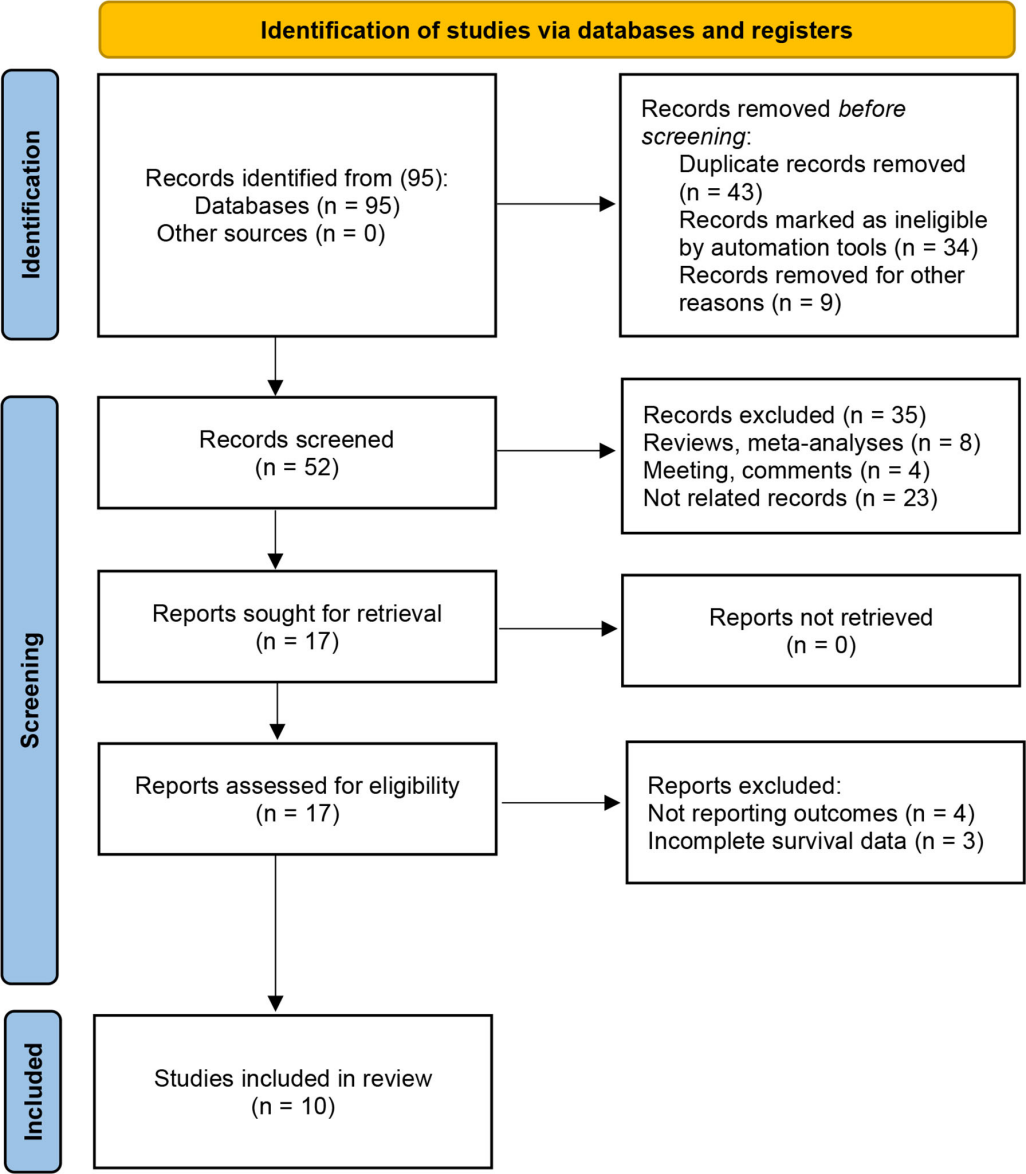
Flow diagram of the study selection process.

A detailed flowchart of the study selection process is presented in **Figure 1**. A computer search initially identified 95 studies, and 52 studies remained after duplicate publications were removed. After having read the titles and abstracts, 35 articles were excluded. Full texts of 17 studies were then reviewed, with 7 studies excluded. Finally, 10 studies that comprised 7,087 patients were included in this meta-analysis (9–13, 16–20). All the included studies have been published within the last 3 years (2019–2021); five studies focused on NMIBC (11, 16, 17, 19, 20) and the other five studies on MIBC (9, 10, 12, 13, 18). Notably, all of the 10 studies were retrospective, and six of them had been conducted in China (9, 11, 12, 16, 19, 20), two in Europe and United States (10, 17), one in Turkey (13), and one in Japan (18). The sample size in the included studies ranged from 79 to 4,335 patients, with the median patient age ranging from 53.6 to 73 years, and the cutoff values of SII ranged from 276 to 896. Seven studies reported the association between SII and OS (9, 10, 12, 13, 16–18), four between SII and CSS (10, 16–18), and six investigated associations between SII and RFS (10–12, 17, 19, 20). Additionally, six studies provided data on the association between SII and clinicopathological characteristics of patients (9, 10, 16–18, 20). None of the studies had an NOS score below 7, indicating that the overall quality of the included studies was high. The main features of the nine included studies are presented in **Table 1**.

**Table 1:** Baseline characteristics of include studies and methodological assessment.

Author	Year	Country	Study design	Tumor type	Treatment	Sample size	Age (years)	Cutoff value	Analysis method	Survival analysis	Follow-up(months)	Qualityscore
Zhang et al. (9)	2019	China	Retrospective	MIBC	RC	139	Median 67 (29–87)	507	Multivariate	OS	NR	7
Bi et al. (16)	2020	China	Retrospective	NMIBC	TURBT	298	Median 71 (34–89)	467	Multivariate	OS, CSS	Median 108 (5–191)	7
Tang et al. (12)	2020	China	Retrospective	MIBC	RC	79	Mean 53.6 ± 7.5	547	Univariate	OS, RFS	Median 31	7
Yılmaz et al. (13)	2020	Turkey	Retrospective	MIBC	RC	152	Median 66 (43–88)	768	Multivariate	OS	Median 16 (1–209)	7
Grossmann et al. (10)	2021	Multi-country	Retrospective	MIBC	RC	4,335	Median 67 (60–73)	610	Multivariate	OS, CSS, RFS	Median 42 (18–85)	8
Katayama et al. (17)	2021	Multi-country	Retrospective	NMIBC	TURBT	1,117	Median 67 (58–74)	580	Multivariate	OS, CSS, RFS	Median 64 (26–110)	8
Ke et al. (11)	2021	China	Retrospective	NMIBC	TURBT	184	61.9 ± 10.6	440	Multivariate	RFS	Median 15 (5–63)	7
Yamashita et al. (18)	2021	Japan	Retrospective	MIBC	RC	237	Median 73 (67–79)	438	Multivariate	OS, CSS	Median 38 (17–64)	8
Wang et al. (19)	2021	China	Retrospective	NMIBC	TURBT	330	Mean 65.7 ± 11.9	896	Multivariate	RFS	Mean 21.4 ± 17.2	7
Zhao et al. (20)	2021	China	Retrospective	NMIBC	TURBT	216	Median 59 (25–87)	276	Multivariate	RFS	Median 59.4 (2–89)	7

MIBC, muscle-invasive bladder cancer; NMIBC, non-muscle-invasive bladder cancer; OS, overall survival; RC, radical cystectomy; TURBT, transurethral resection of bladder tumor; CSS, cancer-specific survival; RFS, recurrence-free survival; NR, not reported.

### Prognostic Significance of Systemic Immune-Inflammation Index on Overall Survival in Patients With Bladder Cancer

**Figure 2:**
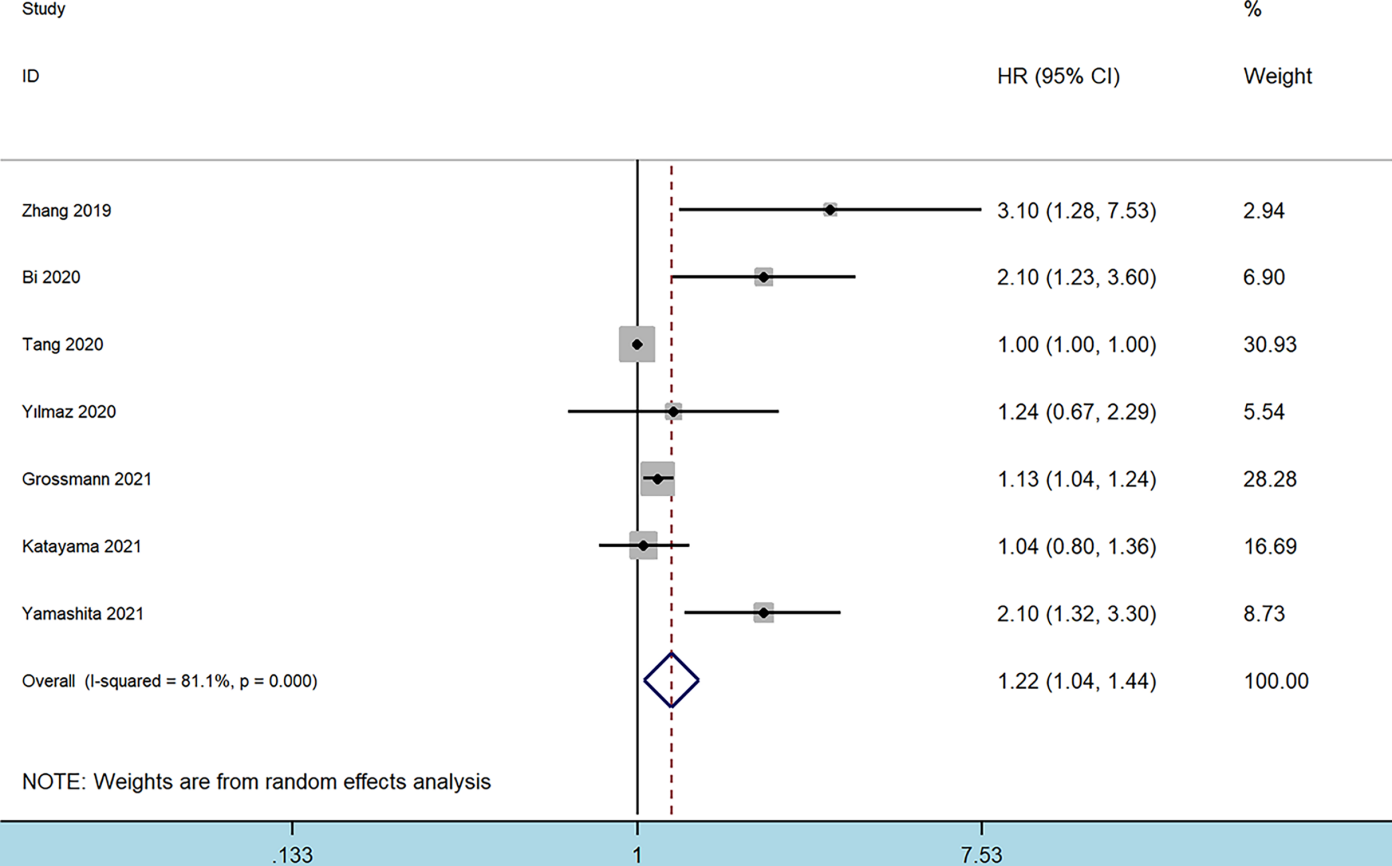
Forest plot of the association between systemic immune-inflammation index and overall survival in patients with bladder cancer.

Seven studies involving 6,357 patients reported an association between preoperative SII and OS in patients with BC (9, 10, 12, 13, 16–18). The pooled analysis indicated that patients with an increased preoperative SII had a significantly worse OS (HR = 1.22, 95% CI 1.04–1.44, *p* = 0.013), with significant heterogeneity between studies (I^2^ = 81.1%, *p* < 0.001) (**Figure 2** and **Table 2**). We also conducted subgroup analyses by ethnicity, tumor type, cutoff value, and sample size. High SII was also significantly associated with poor OS in subgroups with Caucasian ethnicity (*p* = 0.006), patients with MIBC (*p* = 0.043), patient sample size >200 (*p* = 0.031), and cutoff value >550 (*p* = 0.006) (**Table 2**).

**Table 2:** Subgroup analyses of OS, CSS, and RFS.

Outcome	Variable	No. of studies	Model	HR (95% CI)	*p*	Heterogeneity	
						I^2^ (%)	*p*
**OS**	All	7	Random	1.22 (1.04, 1.44)	0.013	81.1	0.000
Ethnicity	Asian	4	Random	1.79 (1.00, 3.20)	0.050	87.4	0.000
	Caucasian	3	Random	1.12 (1.03, 1.22)	0.006	0.0	0.802
Tumor type	MIBC	5	Random	1.20 (1.01, 1.43)	0.043	83.5	0.000
	NMIBC	2	Random	1.42 (0.72, 2.82)	0.315	81.2	0.021
Sample size	>200	4	Random	1.38 (1.03, 1.85)	0.031	75.4	0.007
	≤200	3	Random	1.36 (0.79, 2.33)	0.272	70.3	0.035
Cutoff value	>550	3	Random	1.12 (1.03, 1.22)	0.006	0.0	0.802
	≤550	4	Random	1.79 (1.00, 3.20)	0.050	87.4	0.000
**CSS**	All	4	Random	1.68 (1.14, 2.47)	0.009	73.6	0.011
Ethnicity	Asian	2	Random	1.83 (1.28, 2.61)	0.001	0.0	0.707
	Caucasian	2	Random	1.64 (0.78, 3.43)	0.192	84.6	0.011
Tumor type	MIBC	2	Random	1.42 (0.88, 2.31)	0.153	70.5	0.066
	NMIBC	2	Random	2.02 (0.95, 2.93)	0.064	20.8	0.077
Sample size	>200	3	Random	1.70 (1.02, 2.85)	0.043	78.9	0.009
	≤200	1	–	1.72 (1.06, 2.78)	0.028	–	–
Cutoff value	>550	2	Random	1.64 (0.78, 3.43)	0.192	84.6	0.707
	≤550	2	Random	1.83 (1.28, 2.61)	0.001	0.0	0.707
**RFS**	All	6	Random	1.29 (1.03, 1.61)	0.027	90.2	0.000
Ethnicity	Asian	4	Random	1.35 (0.76, 2.41)	0.311	92.9	0.000
	Caucasian	2	Random	1.18 (1.00, 1.40)	0.055	29.4	0.234
Tumor type	MIBC	2	Random	1.05 (0.94, 1.18)	0.405	79.9	0.026
	NMIBC	4	Random	1.48 (0.77, 2.84)	0.236	89.2	0.000
Sample size	>200	4	Random	1.40 (0.77, 2.54)	0.266	91.2	0.000
	≤200	2	Random	1.14 (0.83, 1.56)	0.416	79.0	0.029
Cutoff value	>550	3	Random	1.84 (0.97, 3.52)	0.064	92.8	0.000
	≤550	3	Random	1.00 (0.70, 1.43)	0.994	77.4	0.012

MIBC, muscle-invasive bladder cancer; NMIBC, non-muscle-invasive bladder cancer; OS, overall survival; CSS, cancer-specific survival; RFS, recurrence-free survival; HR, hazard ratio; CI, confidence interval.

### Prognostic Significance of Systemic Immune-Inflammation Index on Cancer-Specific Survival in Patients With Bladder Cancer

**Figure 3:**
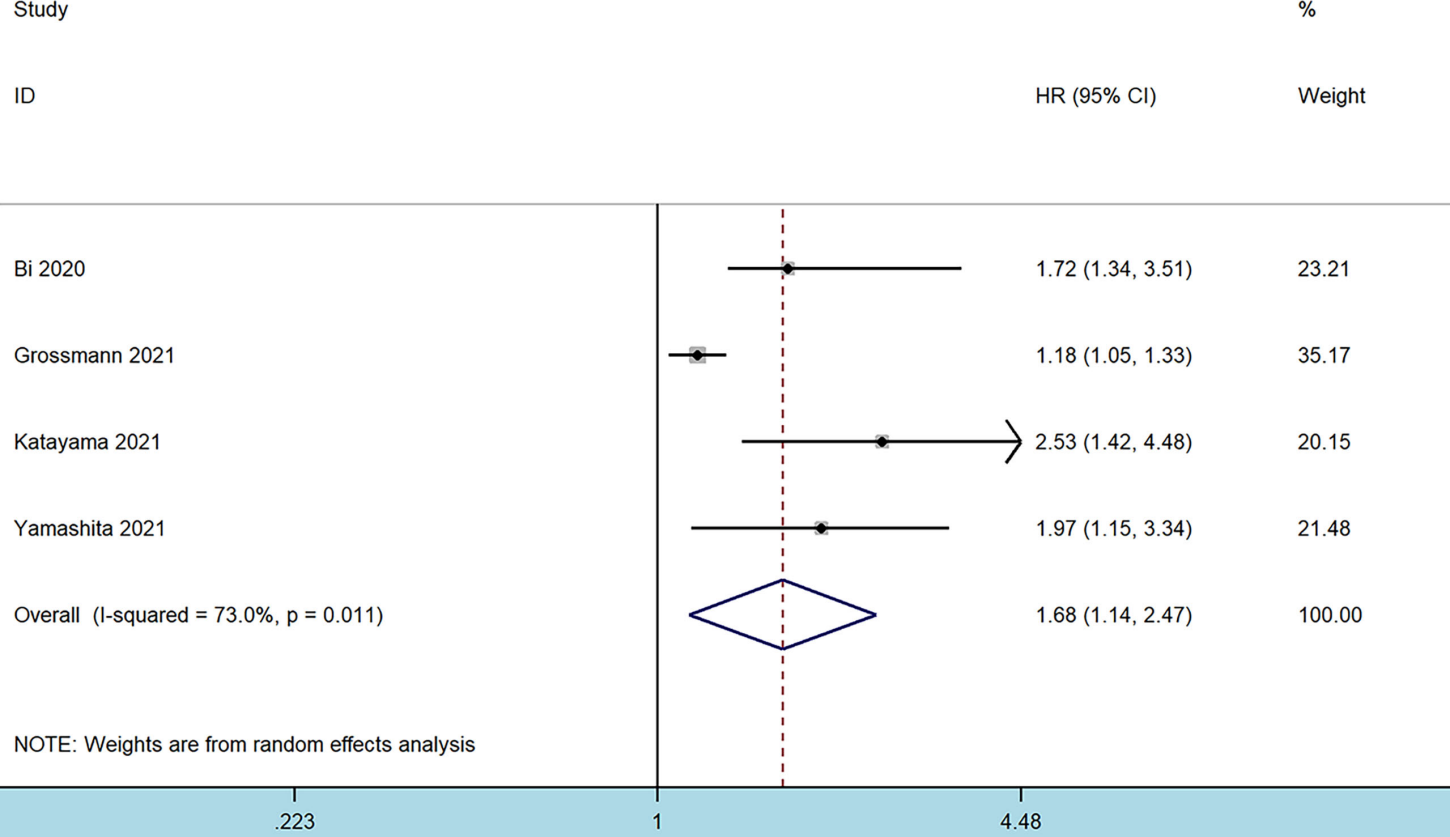
Forest plot of the association between systemic immune-inflammation index and cancer-specific survival in patients with bladder cancer.

Four studies comprising 5,987 patients reported on the prognostic effect of preoperative SII on CSS in BC patients (10, 16–18). The pooled analysis demonstrated that higher preoperative SII in BC patients was an independent predictor of CSS (HR = 1.68, 95% CI 1.14–2.47, *p* = 0.009), with significant heterogeneity (I^2^ = 73.6%, *p* = 0.011) (**Figure 3** and **Table 2**). Furthermore, an elevated SII was significantly associated with inferior CSS in patients with Asian ethnicity (*p* = 0.001), sample size ≤200 (*p* = 0.028), and cutoff values ≤550 (*p* = 0.001) (**Table 2**).

### Prognostic Significance of Systemic Immune-Inflammation Index on Recurrence-Free Survival in Patients With Bladder Cancer

**Figure 4:**
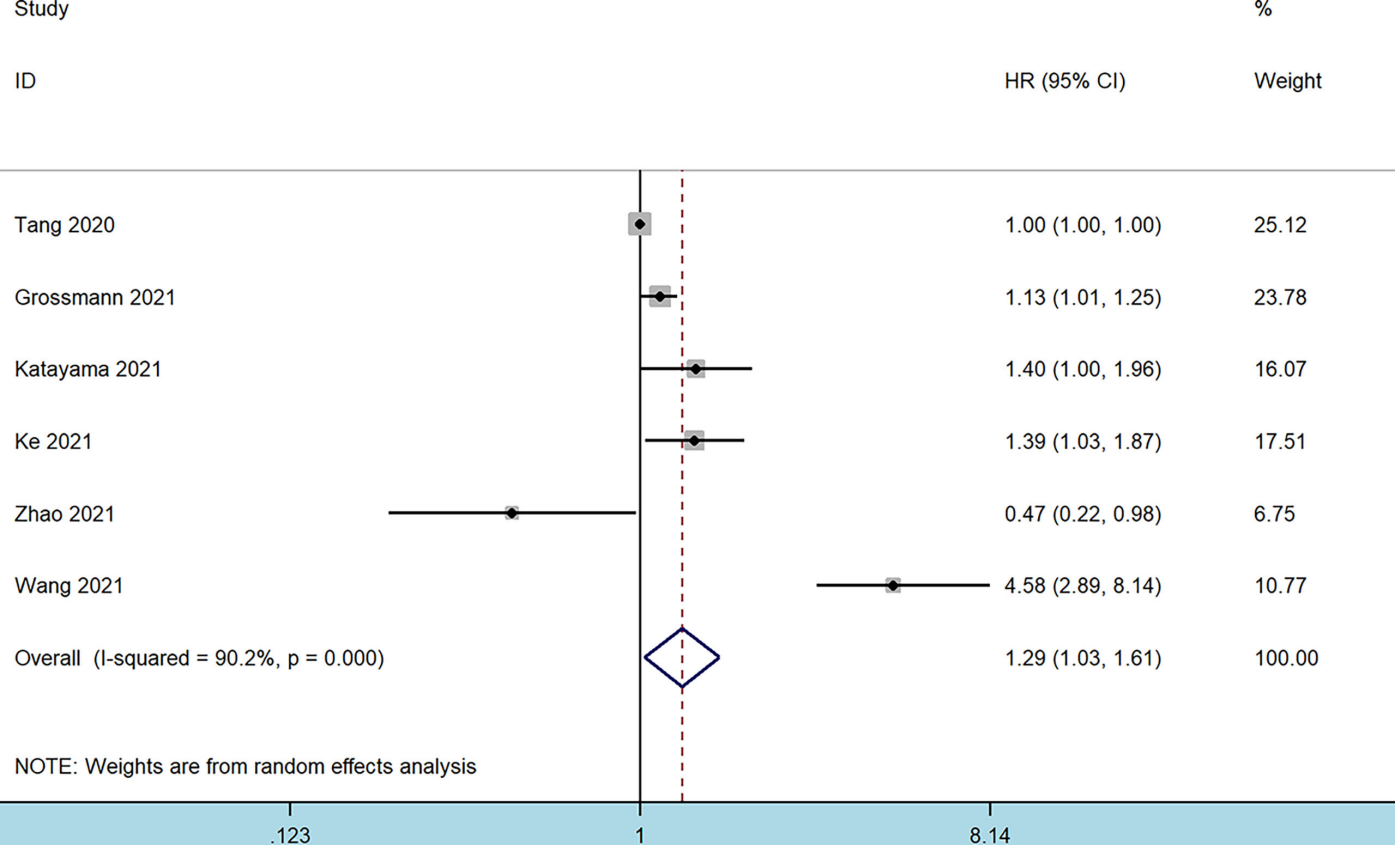
Forest plot of the association between systemic immune-inflammation index and recurrence-free survival in patients with bladder cancer.

Data for RFS analysis were extracted from six studies comprising 5,931 patients (10–12, 17, 20). The pooled analysis revealed that patients with an elevated preoperative SII had an inferior RFS (HR = 1.29, 95% CI 1.03–1.61, *p* = 0.027). Significant heterogeneity among these studies was also observed (I^2^ = 92.9%, *p* < 0.001) (**Figure 4** and **Table 2**). However, a higher SII was not associated with a worse RFS regardless of the ethnicity, tumor type, sample size, or cutoff value (**Table 2**).

### Correlations Between Systemic Immune-Inflammation Index and Clinicopathological Factors in Bladder Cancer

**Figure 5:**
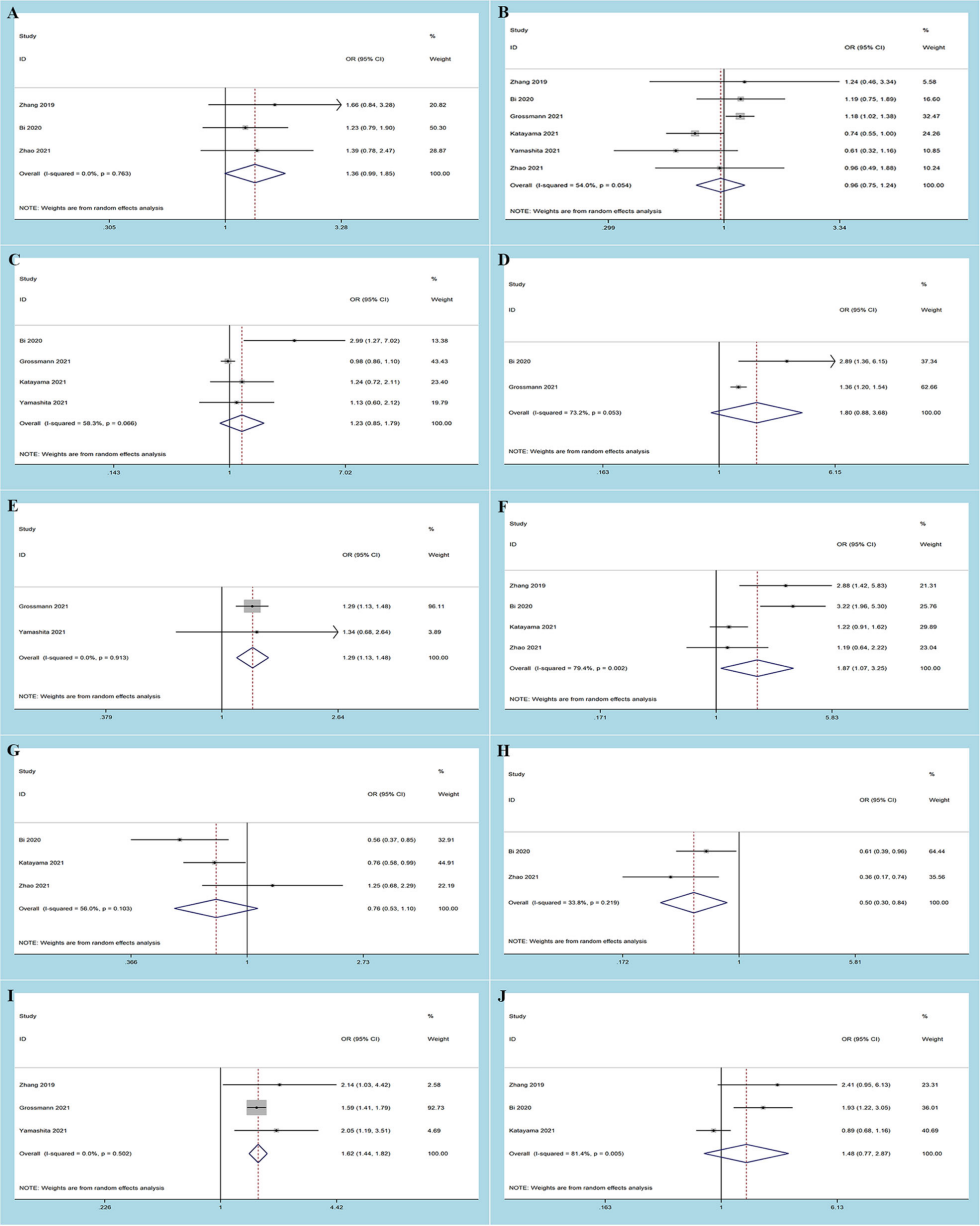
Forest plots of the association between systemic immune-inflammation index and clinicopathological features in bladder cancer: **(A)** age (old vs. young); **(B)** sex (male vs. female); **(C)** concomitant carcinoma *in situ* (yes vs. no); **(D)** lymph node involvement (yes vs. no); **(E)** lymphovascular invasion (yes vs. no); **(F)** tumor size (≥3 cm vs. <3 cm); **(G)** tumor number (unifocal vs. multifocal); **(H)** tumor differentiation (poor vs. well); **(I)** tumor stage [muscle-invasive bladder cancer (MIBC): ≥T3 vs. <T3; **(J)** tumor stage non-muscle-invasive bladder cancer (NMIBC): T1 vs. Tis-Ta].

Six studies reported the relationship between SII and clinicopathological features of BC (9, 10, 16–18, 20), which included age (old vs. young), sex (male vs. female), concomitant carcinoma *in situ* [(CIS) yes vs. no], lymph node involvement [(LNI) yes vs. no], lymphovascular invasion [(LVI) yes vs. no], tumor size (≥3 cm vs. <3 cm), tumor number (unifocal vs. multifocal), tumor differentiation (poor vs. well), and tumor stage (MIBC: ≥T3 vs. <T3; NMIBC: T1 vs. Tis-Ta). The pooled analysis showed that an increased preoperative SII correlated with poor tumor differentiation (OR = 1.98, 95% CI 1.20–2.29, *p* = 0.008), advanced tumor stage of MIBC (OR = 1.62, 95% CI 1.44–1.83, *p* < 0.001), presence of LNI (OR = 1.30, 95% CI 1.13–1.48, p < 0.001), and tumor size ≥3 cm (OR = 1.87, 95% CI 1.07–3.25, *p* = 0.027). However, no significant association was observed between SII and age (OR = 1.36, 95% CI 1.00–1.85, *p* = 0.439), sex (OR = 0.97, 95% CI 0.75–1.24, *p* = 0.780), concomitant CIS (OR = 1.23, 95% CI 0.85–1.79, *p* = 0.269), LVI (OR = 1.80, 95% CI 0.89–3.68, *p * = 0.107), tumor number (OR = 0.77, 95% CI 0.53–1.10, *p* = 0.146), or tumor stage in NMIBC (OR = 1.48, 95% CI 0.77–2.87, *p* = 0.243) (**Table 3** and **Figure 5**).

**Table 3:** Correlations of SII and clinicopathological characteristics in patients with bladder cancer.

Characteristics	No. of studies	No. of patients	Effects model	OR (95% CI)	*p*	Heterogeneity	
						I^2^ (%)	*p*
Age (old vs. young)							
Binary variables	3	5,689	Random	1.36 (1.00, 1.85)	0.439	70.2	0.035
Sex (male vs. female)	6	6,431	Random	0.97 (0.75, 1.24)	0.780	54.0	0.054
Concomitant CIS (yes vs. no)	4	6,070	Random	1.23 (0.85, 1.79)	0.269	58.3	0.066
LNI (yes vs. no)	2	4,572	Random	1.30 (1.13, 1.48)	0.0	0.0	0.913
LVI (yes vs. no)	2	4,722	Random	1.80 (0.89, 3.68)	0.107	73.2	0.053
Tumor size (≥3 cm vs. <3 cm)	4	1,859	Random	1.87 (1.07, 3.25)	0.027	79.4	0.002
Tumor number (unifocal vs. multifocal)	3	1,720	Random	0.77 (0.53, 1.10)	0.146	56.0	0.103
Tumor differentiation (poor vs. well)	2	603	Random	1.98 (1.20, 2.29)	0.008	33.8	0.219
Tumor stage							
MIBC (≥T3 vs. < T3)	3	4,711	Random	1.62 (1.44, 1.83)	0.0	0.0	0.502
NMIBC (T1 vs. < Tis-Ta)	3	1,719	Random	1.48 (0.77, 2.87)	0.243	81.4	0.005

SII, systemic immune-inflammation index; CIS, carcinoma in situ; LNI, lymph node involvement; LVI, lymphovascular invasion; MIBC, muscle-invasive bladder cancer; NMIBC, non-muscle-invasive bladder cancer; OR, odds ratio; CI, confidence interval.

### Sensitivity Analysis

Sensitivity analyses were conducted to evaluate the reliability of pooled HRs for OS, CSS, and RFS (**Figure S1**). The leave-one-out test showed no significant change in the overall HR estimates for these survival outcomes, suggesting that the results of this meta-analysis were relatively stable and reliable.

### Publication Bias

We used Begg’s test to assess the publication bias in included studies. Although the results of this test were not statistically significant (OS: *p* = 0.764, CSS: *p* = 0.089, RFS: *p* = 1.000), visual examination of Begg’s funnel plot showed asymmetry, which increased the possibility of potential publication bias (**Figure S2**).

## Discussion

The high postoperative recurrence rate for patients with BC often affects the patient’s quality of life, which gives researchers the impetus to identify and analyze relevant risk factors affecting tumor recurrence and to develop individualized treatment and follow-up plans for patients with differing recurrence risks. Although histopathological types, histological grades, and pathological stages have been used to predict relapse and progression in patients with BC (3, 21), the heterogeneity of prognosis among patients with similar clinical presentations suggests that more reliable indicators are needed to identify high-risk patients. Recently, an inflammation-related SII obtained from blood samples was shown to be a novel biomarker for predicting the therapeutic efficacy or prognosis among patients with different types of cancers, regardless of the treatment regimen (8, 22). However, the prognostic value of this index in BC is still controversial. Huang et al. (22) performed a systematic review to analyze the prognostic value of SII in urological cancers, and they reported that BC patients with an elevated SII had poorer OS. However, they only analyzed two studies, which greatly reduced the reliability of their conclusions. With the successive publications of relevant studies, a comprehensive investigation of the potential value of SII in terms of clinicopathological features and the prognosis for BC patients is warranted.

To elucidate the role of the SII in the prognosis of BC, data were pooled from 10 studies involving 7,087 patients in the present meta-analysis. The pooled analysis suggested that patients with an increased preoperative SII had inferior OS, CSS, and RFS outcomes. Subgroup analysis also indicated that elevated SII was related to worse OS in MIBC. In addition, high preoperative SII was significantly associated with poor tumor differentiation, high tumor stage of MIBC, presence of LNI, and tumor size ≥3 cm. Taken together, SII could be a potential prognostic biomarker for BC.

Furthermore, host inflammatory responses in cancer have received increasing attention over the past 20 years. Growing evidence suggests that immune cells play an important role in the inflammation process, leading to the production of cytokines and chemokines that accelerate tumor growth, neovascularization, and metastasis (23, 24). A complex balance between inflammatory cells and inflammation-related substances might affect the type of peripheral circulating cells (18). Neutrophils have been linked to tumor progression (25). These cells can infiltrate into the TME and become tumor-related neutrophils, releasing chemical and cytokine factors related to tumor proliferation and metastasis such as vascular endothelial growth factor, elastase, and matrix metalloproteinases (26, 27). Similarly, platelets could release vascular endothelial growth factors, which promote the formation of blood vessels associated with the formation of new tumors and cause adhesion of tumor cells to the vascular wall. This further leads to the proliferation and metastasis of tumor cells (28). However, as the most important immune cells in the body, lymphocytes mainly induce the lysis and apoptosis of target cells and play an antitumor role (29). The lower the level of lymphocytes, the poorer the immune capability of patients; furthermore, the increase of neutrophils could inhibit the activation of T lymphocytes, leading to the decrease of antitumor ability of the body (30). The inflammation, therefore, is closely related to the TME and tumor progression.

In this context, some biomarkers based on peripheral blood cell counts have been proposed to predict tumor prognosis. Nevertheless, when only one or two parameters are involved, these predictors become unstable and are often susceptible to other confounding factors (31). SII is calculated in terms of the neutrophil count × platelet count/lymphocyte count, and it contains three types of inflammatory cells, which objectively reflect the balance between inflammation and the host immune response (20). A higher SII corresponds to higher platelet/neutrophil and/or lower lymphocyte counts. Thus, an elevated SII indirectly symbolizes the deficiency of immune function and the enhancement of tumor aggressiveness in cancer patients (32). As a composite immune index, SII may have better predictive power than parameters such as NLR, PLR, and LMR. Furthermore, the measurement of SII is simple, inexpensive, and reproducible, which makes it a promising biomarker for BC prognosis.

Several previous meta-analyses have investigated the prognostic role of SII in solid tumors. In a pooled analysis of data from 22 studies, Yang et al. (8) found that increased SII may be a reliable prognostic factor for worse OS in various malignancies. Qiu et al. (33) provided evidence that high pretreatment SII was significantly related to worse OS (HR = 1.40, *p* = 0.010) and several clinical features such as advanced tumor stage, positive node metastasis, and larger tumor size in gastric cancer (GC). They reported that SII can be monitored to guide prognosis and provide useful information about the risk of disease progression in GC. One recent study also indicated that a higher SII was independently related to inferior survival outcomes in renal cell carcinoma patients and that elevated SII suggested more aggressive disease (34). A baseline SII was also found to be useful in clinical practice to stratify patients with metastatic urothelial carcinoma (35). Moreover, Katayama et al. (17) also reported that SII could help improve the decision-making process regarding adjuvant therapy for patients at intermediate risk for NMIBC. The findings of this analysis indicated that SII had prognostic efficiency in terms of OS, CSS, and RFS in patients with BC, which was consistent with findings about other cancer types. We also indicated a significant association between higher SII and invasive pathologic features of BC. Based on these findings and those of other related studies, SII could be considered as an effective prognostic marker for BC that could facilitate risk stratification in patients with BC and help develop appropriate clinical treatment strategies.

Although this analysis provides additional substantial evidence regarding the prognostic value of preoperative SII in BC patients, this study also had several limitations. First, the included studies were retrospectively designed; therefore, inherent structural biases associated with retrospective studies may have contributed to inter-study heterogeneity. Second, although all patients with BC had been treated surgically, postoperative adjuvant therapy strategies and pathological indications are known to vary across institutions, which may have resulted in different survival outcomes. Third, different cutoff values and measurement methods of SII in the included studies may have led to bias in the results. Fourth, due to heterogeneity between the studies, an optimal SII cutoff value to predict postoperative outcomes in patients with BC cannot be determined presently. To address these limitations, further multicenter prospective studies with larger sample sizes are needed.

## Conclusions

The present evidence indicates that a higher preoperative SII is associated with worse OS, CSS, and RFS in patients with BC. Furthermore, we demonstrate that elevated SII is independently correlated with adverse pathological features. SII as an effective prognostic indicator may have a crucial role in improving clinical decision-making during BC treatment. However, further study on the role of SII in BC prognosis is required before its widespread use in clinical practice.

## Data Availability Statement

The original contributions presented in the study are included in the article/**Supplementary Material**. Further inquiries can be directed to the corresponding authors.

## Author Contributions

Conception/design: QW and TL. Collection and/or assembly of data: JL, DC, and YH. Data analysis and interpretation: JL, LL, QX, and DT. Article writing: JL and DC. Final approval of article: all authors.

## Funding

This study was supported by the National Natural Science Foundation of China (Grant Number 81974098, 82000721); Post-Doctor Research Project, West China Hospital, Sichuan University (Grant Number 2019HXBH089); and Health Commission of Sichuan province (Grant Number 20PJ036).

## Conflict of Interest

The authors declare that the research was conducted in the absence of any commercial or financial relationships that could be construed as a potential conflict of interest.

## Publisher’s Note

All claims expressed in this article are solely those of the authors and do not necessarily represent those of their affiliated organizations, or those of the publisher, the editors and the reviewers. Any product that may be evaluated in this article, or claim that may be made by its manufacturer, is not guaranteed or endorsed by the publisher.
