# Supplementary material for: The Prognostic and Clinicopathological Significance of Systemic Immune-Inflammation Index in Bladder Cancer
Source: Front Immunol. 2022 Apr 28;13:865643. doi: 10.3389/fimmu.2022.865643 (PMC9097688; doi:10.3389/fimmu.2022.865643)

**Figure S2.** Detection of publication bias in meta-analyses of (**A**) overall survival, (**B**) cancer-specific survival, and (**C**) recurrence-free survival.


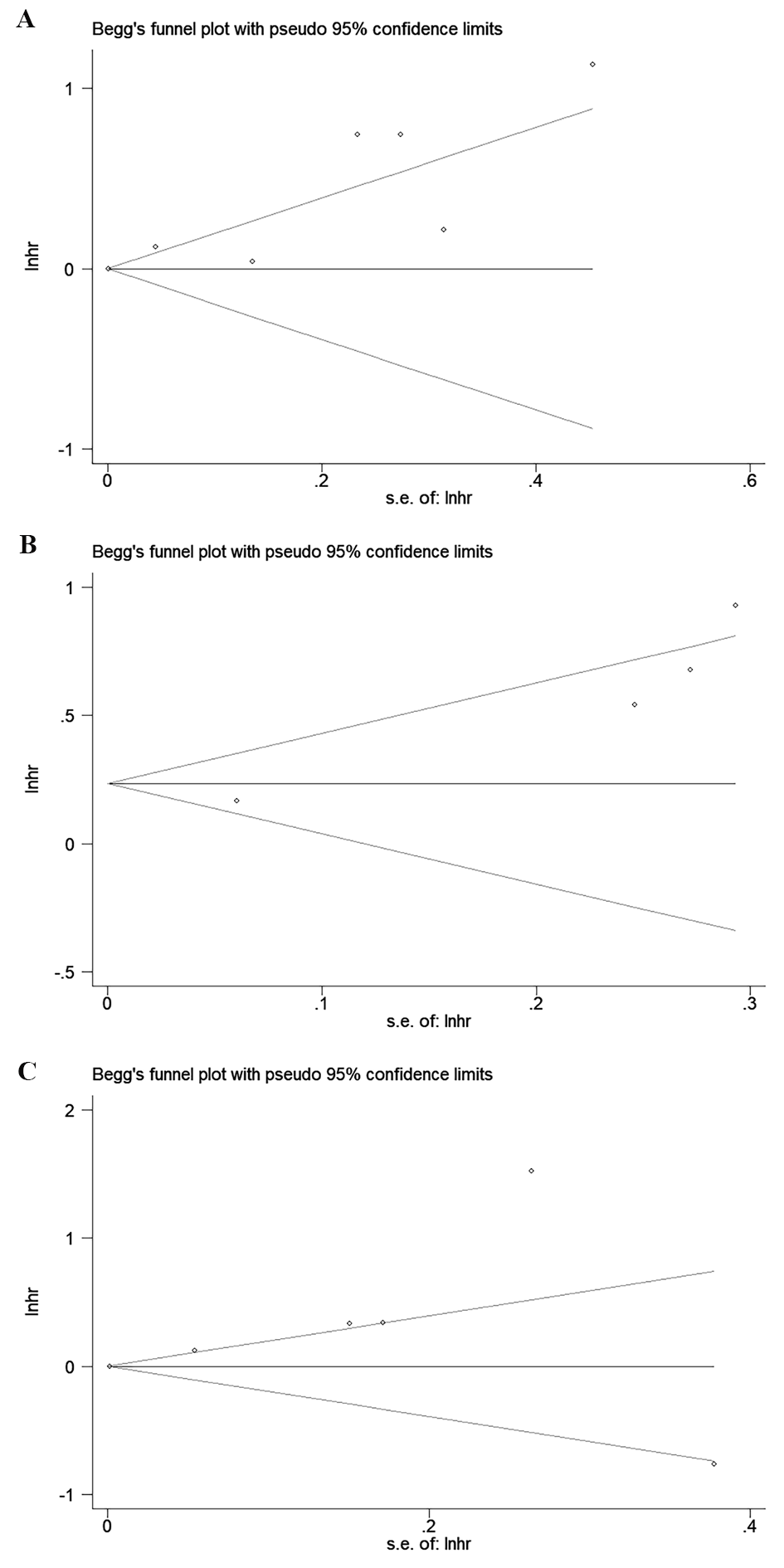

Supplement: Supplementary file 1 [file DataSheet_1.zip › Supplementary figure/Figure S2.docx]
